# Nitro Capsaicin Suppressed Microglial Activation and TNF-α-Induced Brain Microvascular Endothelial Cell Damage

**DOI:** 10.3390/biomedicines10112680

**Published:** 2022-10-23

**Authors:** Sopana Jamornwan, Tanida Chokpanuwat, Kwanchanok Uppakara, Thanet Laorob, Uthai Wichai, Pimonrat Ketsawatsomkron, Witchuda Saengsawang

**Affiliations:** 1 Department of Physiology, Faculty of Science, Mahidol University, Bangkok 10400, Thailand; 2 Chakri Naruebodindra Medical Institute, Faculty of Medicine, Ramathibodi Hospital, Mahidol University, Samut Prakan 10540, Thailand; 3 Department of Chemistry, Faculty of Science, Naresuan University, Phitsanulok 65000, Thailand; 4 Department of Basic Biomedical Sciences, Dr. William M. Scholl College of Podiatric Medicine, Rosalind Franklin University of Medicine and Science, North Chicago, IL 60064, USA

**Keywords:** neuroinflammation, microglial activation, brain microvascular endothelial cell damage, nitro capsaicin, capsaicin

## Abstract

Chronically activated microglia and brain vascular damage are major causes of neuroinflammation. The aim of this study was to determine the anti-inflammatory effects of nitro capsaicin, a newly modified capsaicin with less irritating characteristics, against microglial activation and brain microvascular endothelial cell damage. Using the SIMA9 microglia cell line, we found that nitro capsaicin reduced nitric oxide (NO) production in LPS-activated microglia better than its parent compound, capsaicin. Nitro capsaicin also decreased the expression of proinflammatory cytokines (TNF-α, IL-1β, and IL-6) and enhanced the levels of anti-inflammatory factors, IL-4 and IL-10, both at the mRNA and protein levels. In the TNF-α-induced vascular damage model, nitro capsaicin decreased expression and secretion of the proinflammatory cytokines IL-1β and IL-6. Phosphorylated NF-κB p65, a key transcription factor that stimulates the signaling of inflammatory pathways, was also reduced in the presence of nitro capsaicin, suggesting that the anti-inflammatory effects of nitro capsaicin were created through reducing NF-κB activation. Together, we concluded that nitro capsaicin has the potential to be further developed as an anti-neuroinflammatory agent.

## 1. Introduction

Neuroinflammation plays an important role in the development and progression of neurodegenerative diseases such as Alzheimer’s disease (AD) and Parkinson’s disease (PD) [1,2,3]. Several cell types in the central nervous system have been shown to play a role in neuroinflammation, including microglia and brain capillary endothelial cells. Previous studies have reported that the prolonged neuroinflammation that occurs due to chronic microglial activation is involved with the development and progression of AD pathological risk [4,5,6,7]. Furthermore, several pathological hallmarks of AD can directly damage brain capillary endothelial cells and promote blood–brain barrier (BBB) disruption, which has been reported as an early biomarker of cognitive impairment [8,9]. Previous studies have reported that excessive and chronic inflammation induced by microglial activation can promote the secretion of inflammatory mediators, such as cytokines/chemokines and nitric oxide (NO), which further contributes to the damage of neuronal and other brain cells [10,11,12]. Furthermore, several inflammatory factors that are released from the damaged BBB can directly damage neurons and other cell types in the brain, leading to enhance neuroinflammation [13,14,15,16,17]. Therefore, modulating neuroinflammation mediated through microglial activation and brain endothelial capillary damage represents a new target for preventing neurodegeneration and disease progression.

Capsaicin, the main pungent substance in hot chili peppers, belongs to the genus *Capsicum* and the family *Solanaceae*. Capsaicin has received significant attention due to its regular use in food and health-promoting effects. It has also been shown to exert powerful anti-inflammatory effects and to improve behavioral impairment in several neurodegenerative models. For example, capsaicin has been reported to prevent Aβ-induced hippocampal damage in the mouse model of AD [18], to prevent nigrostriatal dopamine neuronal damage, and to improve motor behavioral impairment in LPS and MPTP damage to the substantia nigra [19,20]. However, the exposure to a high dose of capsaicin for a prolonged time can promote irritation due to its pungent characteristics, limiting its usage in humans [21,22,23,24]. Therefore, new compounds with fewer irritation characteristics have been recently developed and modified from the original compound (capsaicin). A recent study has reported that the substitution of the methoxy group (OCH_3_) with a nitrogen dioxide group (NO_2_) in the head section of capsaicin yields the new modified compound nitro capsaicin. Nitro capsaicin is less irritative, as evidenced by an irritation test using normal human dermal fibroblasts during process synthesis [25]. Moreover, a previous study has reported that a similar modified capsaicin with an added nitro group can suppress TNF-α production better in comparison to capsaicin in LPS-stimulated peripheral blood mononuclear cells (PBMCs) [26]. However, the anti-inflammatory effects of nitro capsaicin have yet to be determined. In the present study, we investigated the anti-inflammatory properties of nitro capsaicin against LPS-induced microglial activation using a microglial cell line (SIMA9). Additionally, we examined the effects of nitro capsaicin on TNF-α-driven brain microvascular endothelial inflammation using a human cerebral microvascular endothelial cell line (hCMEC/D3). Moreover, the underlying mechanisms of how nitro capsaicin exerts its anti-inflammation were evaluated in this study.

## 2. Materials and Methods

### 2.1. Capsaicin and Nitro Capsaicin Material

The processed syntheses of the original compound (capsaicin) and modified compound (nitro capsaicin) were conducted as previously described [25]. Capsaicin and nitro capsaicin were provided by Assistant Professor Uthai Wichai (Department of Chemistry, Faculty of Science, Naresuan University, Phitsanulok, Thailand). Both compounds were dissolved in DMSO and stored at −80 °C before use. The final DMSO concentrations were maintained at 0.02% in all experiments.

### 2.2. Cell Culture

SIMA9 cells, a widely used immortalized microglial cell line derived from cerebral cortices of mice, were used for cell culture experiments [27]. This cell line was obtained from the American Type Culture Collection (ATCC^®^ CRL-3265^TM^, Manassas, VA, USA). Cells were cultured in complete DMEM/F12 (Gibco, Carlsbad, CA, USA) with 10% (*v*/*v*) FBS (Hyclone Laboratories Inc., Logan, UT, USA), 5% (*v*/*v*) horse serum (Gibco, Carlsbad, CA, USA), and 1% (*v*/*v*) Penicillin-Streptomycin (Gibco, Carlsbad, CA, USA). Cells were passaged with a subculturing solution containing 1 mM EDTA, 1 mM EGTA, and 1 mg/mL glucose. Human cerebral microvascular endothelial cells (hCMEC/D3) were used in this study. hCMEC/D3 cells have been widely used to develop an in vitro model of the human BBB [28]. This cell line was purchased from MilliporeSigma (#SCC066, Burlington, MA, USA). hCMEC/D3 cells were cultured on 5–10 µg/cm^2^ of collagen type I (Gibco, Carlsbad, CA, USA) and maintained in Endothelial Cell Growth Medium MV2 (PromoCell, Heidelberg, Germany), 5% (*v*/*v*) FBS, and 1% (*v*/*v*) Penicillin-Streptomycin. hCMEC/D3 cells were subcultured with 0.05% (*v*/*v*) Trypsin. Both cell lines were incubated with humidified 5% CO_2_ at 37 °C and were plated overnight (24 h) before being used for experiments.

### 2.3. Cell Viability Assay

Cytotoxicity of the compounds on SIMA9 and hCMEC/D3 cells was determined using 3-(4,5-Dimethyl-2-thiazolyl)-2,5-diphenyl-2H-tetrazolium bromide (MTT). In brief, SIMA9 microglial cells were seeded into a 96-well culture plate at 7.5 × 10^3^ cells/well and coated with 0.01 mg/mL of Poly-D-lysine (MilliporeSigma, Burlington, MA, USA). Brain endothelial hCMEC/D3 cells were cultured at a density of 1.5 × 10^4^ cells/well. Once confluent, the cells were treated with different concentrations of the capsaicin compounds for 24 h. After the media were removed, cells were incubated with 0.5 mg/mL MTT solution (MilliporeSigma, Burlington, MA, USA) for 3 h at 37 °C. After MTT incubation, the supernatant was gently removed, and DMSO (MilliporeSigma, Burlington, MA, USA) was added to each well to dissolve the formazan crystals. The absorbance intensity was measured at 570 nm using a microplate reader (Multiskan GO, Thermo Fisher Scientific, Waltham, MA, USA) to determine cell viability.

### 2.4. Nitric Oxide Assay

Nitric oxide (NO) production was assessed by measuring the amount of nitrite released from microglia using the Griess test. SIMA9 cells (7.5 × 10^3^ cells/well) were pretreated with each capsaicin compound for 1 h and then stimulated with LPS at 10 ng/mL for 24 h. The culture media were collected to detect the levels of NO released. The culture media were subsequently incubated with Griess solutions (MilliporeSigma, Burlington, MA, USA), including 1% (*w*/*v*) Sulfanilamide in 5% (*v*/*v*) H_3_PO_4_ for 10 min and 0.1% (*w*/*v*) NED for 5 min. After incubation, the absorbance intensity at 550 nm was quantified using a microplate reader (Multiskan GO, Thermo Fisher Scientific, Waltham, MA, USA). The NO concentration in each condition was determined from a standard curve of sodium nitrite (NaNO_2_) (MilliporeSigma, Burlington, MA, USA).

### 2.5. Reverse-Transcription Polymerase Chain Reaction

SIMA9 (2 × 10^5^ cells/well) and hCMEC/D3 cells (3 × 10^5^ cells/well) were seeded in 6-well plates. The cells were treated with each capsaicin compound for 1 h before being incubated with or without 10 ng/mL LPS (SIMA9) and 1 ng/mL TNF-α (hCMEC/D3) for 6 h, respectively. The total RNA concentrations for all conditions were extracted using the TRIzol^®^ reagent (Life technologies corporation, Singapore). The total RNA concentrations were then measured using a NanoDrop^TM^ 2000/2000c Spectrophotometer (Thermo Fisher Scientific, Waltham, MA, USA). Next, complementary DNA (cDNA) was synthesized using the iScript^TM^ Reverse Transcription Supermix (Bio-Rad Laboratories, Hercules, CA, USA) following the manufacturer’s instructions. RT-PCR assay was used to estimate mRNA expression levels using the iTaq^TM^ Universal SYBR^®^ Green Supermix (Bio-Rad Laboratories, Hercules, CA, USA). The relative mRNA expression was calculated using the quantification cycle (Cq) value. The Cq value of the gene of interest was subtracted from the Cq values of the loading control GAPDH to represent mRNA expression levels.

### 2.6. Enzyme-Linked Immunosorbent Assay

To determine the effect of the capsaicin compounds on the secretion of cytokines from SIMA9 microglial and hCMEC/D3 brain endothelial cells, cytokine levels were measured using MILLIPLEX^®^ Mouse and Human Cytokine/Chemokine Magnetic Bead Panels (MilliporeSigma, Burlington, MA, USA), respectively. The SIMA9 (2 × 10^5^ cells/well) and hCMEC/D3 (3 × 10^5^ cells/well) were first cultured into 6-well plates and subsequently pretreated with each compound for 1 h. The cells were then stimulated with 10 ng/mL LPS (SIMA9) and 1 ng/mL TNF-α (hCMEC/D3) for 24 h, respectively. After 24 h, the culture media were collected, and the cytokine concentrations were quantified using MILLIPLEX^®^ MAP Cytokine/Chemokine kits according to the manufacturer’s protocols. The culture media were incubated and shaken overnight with magnetic beads coated with antibodies in 96-black well plates at 4 °C. Next, biotinylated detection antibodies and Streptavidin-Phycoerythrin were added to each well, and they were incubated for 1 h and 30 min, respectively. After washing, Sheath Fluid was incubated and shaken for 5 min. The fluorescence signals were assessed using the Luminex MAGPIX^®^ system (MilliporeSigma, Burlington, MA, USA) and then quantified for cytokine concentrations.

### 2.7. Western Blot

SIMA9 (2 × 10^5^ cells/dish) and hCMEC/D3 cells (3 × 10^5^ cells/dish) were pretreated with each capsaicin compound for 1 h prior to exposure to 10 ng/mL LPS (SIMA9) and 1 ng/mL TNF-α (hCMEC/D3) for 30 min (NF-ĸB) and 24 h (iNOS), respectively. After incubation, total protein lysates were extracted, and the total protein concentration was measured via a BCA assay kit (Thermo Fisher Scientific, Waltham, MA, USA). Proteins in the lysate were separated through sodium dodecyl sulfate-polyacrylamide gel electrophoresis (SDS-PAGE). Resolved proteins were subsequently transferred to Polyvinylidene difluoride (PVDF) membranes, and the non-specific bindings were blocked with 5% (*w*/*v*) non-fat dry milk for 1 h. The membranes were then incubated with a specific primary antibody overnight at 4 °C. The antibodies were used at the following dilutions: anti-iNOS (1:250, Abcam, Cambridge, UK), anti-phospho-NF-κB p65 Ser536 (1:1000, Santa Cruz Biotechnology Inc., Dallas, TX, USA), anti-NF-κB p65 (1:1000, Cell Signaling Technology, Danvers, MA, USA), and anti-GAPDH (1:10,000, Thermo Fisher Scientific, Waltham, MA, USA). The membranes were rinsed 6 times with Tris-buffered saline with Tween-20 (TBS-T) for 10 min and then exposed to HRP-conjugated secondary antibody (Jackson ImmunoResearch Laboratories Inc., West Grove, PA, USA) for 1 h. Next, the membranes were rinsed, and the protein bands were detected using an enhanced chemiluminescence (ECL) reagent (MilliporeSigma, Burlington, MA, USA) for 5 min. The protein expression levels were normalized with GAPDH and quantified using ImageJ software (National Institutes of Health, Bethesda, MD, USA).

### 2.8. Immunofluorescence Staining Assay

hCMEC/D3 cells were cultured at a density of 1.5 × 10^4^ cells/well in a 96-well black culture plate and pretreated with nitro capsaicin for 1 h. The cells were then treated with 1 ng/mL TNF-α for 30 min. Next, the cells were fixed with cold methanol for 10 min at −20 °C before washing with DPBS (Hyclone Laboratories Inc., Logan, UT, USA) for 5 min each 3 times. After that the cells were blocked with 1% (*v*/*v*) BSA (MilliporeSigma, Burlington, MA, USA), 22.52 mg/mL Glycine (MilliporeSigma, Burlington, MA, USA), and 0.1% (*v*/*v*) Tween-20 (MilliporeSigma, Burlington, MA, USA) in DPBS for 45 min at room temperature. Next, the anti-NF-ĸB p65 primary antibody (1:500, Cell Signaling Technology, Danvers, MA, USA) was incubated at 4 °C overnight. After that, the cells were washed for 5 min 3 times before incubation with Alexa Fluor 488 anti-rabbit secondary antibody (Thermo Fisher Scientific, Waltham, MA, USA) at 1:500 and DAPI (Life technologies corporation, Singa-pore) at 1:500 for 1 h at room temperature in the dark condition. Finally, the cells were washed again before being imaged by high-content imaging system (Operetta, PerkinElmer, Waltham, MA, USA). The NF-ĸB translocation that represents the inflammatory cells was calculated and analyzed using Harmony software (PerkinElmer, Waltham, MA, USA).

### 2.9. Statistical Analysis

The data were presented as mean ± SEM from (at least) three independent experiments. The statistical analysis was performed using a one-way analysis of variance (ANOVA) followed by a Tukey’s post hoc test in GraphPad Prism software Version 9.4.1 (GraphPad Software Inc., San Diego, CA, USA). It was considered statistically significant when *p*-values < 0.05.

## 3. Results

### 3.1. Cytotoxic Potency of Nitro Capsaicin in SIMA9 Cells

**Figure 1:**
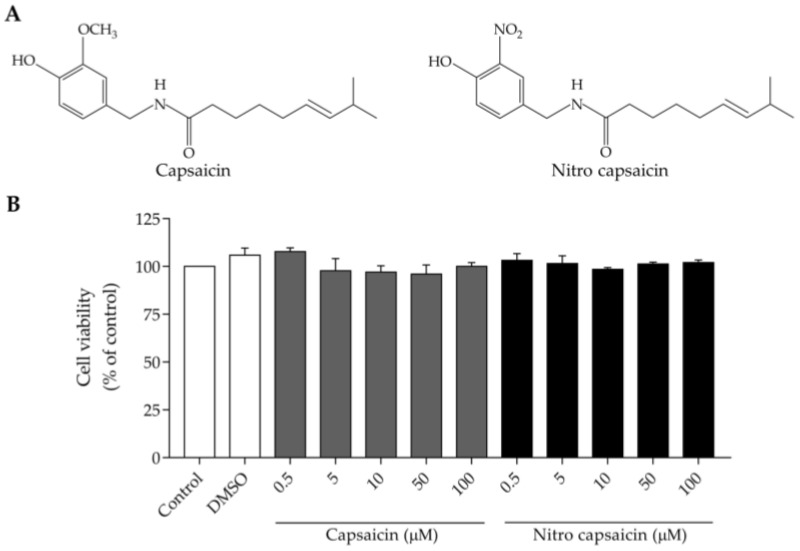
Effect of nitro capsaicin and capsaicin on SIMA9 cell viability. (**A**) Structure of the original compound (capsaicin) and the modified compound (nitro capsaicin); (**B**) Viability of SIMA9 cells after exposure to different concentrations of nitro capsaicin and capsaicin for 24 h (*n* = 3).

Capsaicin has potent anti-inflammatory properties [18,19,20,29,30]; however, high irritation to exposed areas has limited its widespread use [21,22,23,24]. A recent study reported that a modified form of capsaicin, nitro capsaicin, produces less irritation [25]. However, its anti-inflammatory effects have never been investigated. Therefore, we compared the effects of the modified compound (nitro capsaicin) and the original compound (capsaicin) on microglial activation. We first determined the non-toxic concentrations of nitro capsaicin and capsaicin (Figure 1A) on SIMA9 cells using an MTT assay. The cells were incubated with various concentrations of either nitro capsaicin or capsaicin (0.5–100 µM) for 24 h. The matched controls, with the same concentration of DMSO as those treated with capsaicinoid compounds, were included to ensure that the cell viability was not affected by DMSO itself. We observed that treatment with nitro capsaicin or capsaicin did not affect cell viability with the concentrations tested (Figure 1B). Therefore, nitro capsaicin and capsaicin concentrations from 10–100 µM were used in further studies.

### 3.2. Nitro Capsaicin Inhibited Nitric Oxide Production, iNOS Expression, and NF-κB Activation in SIMA9 Cells

**Figure 2:**
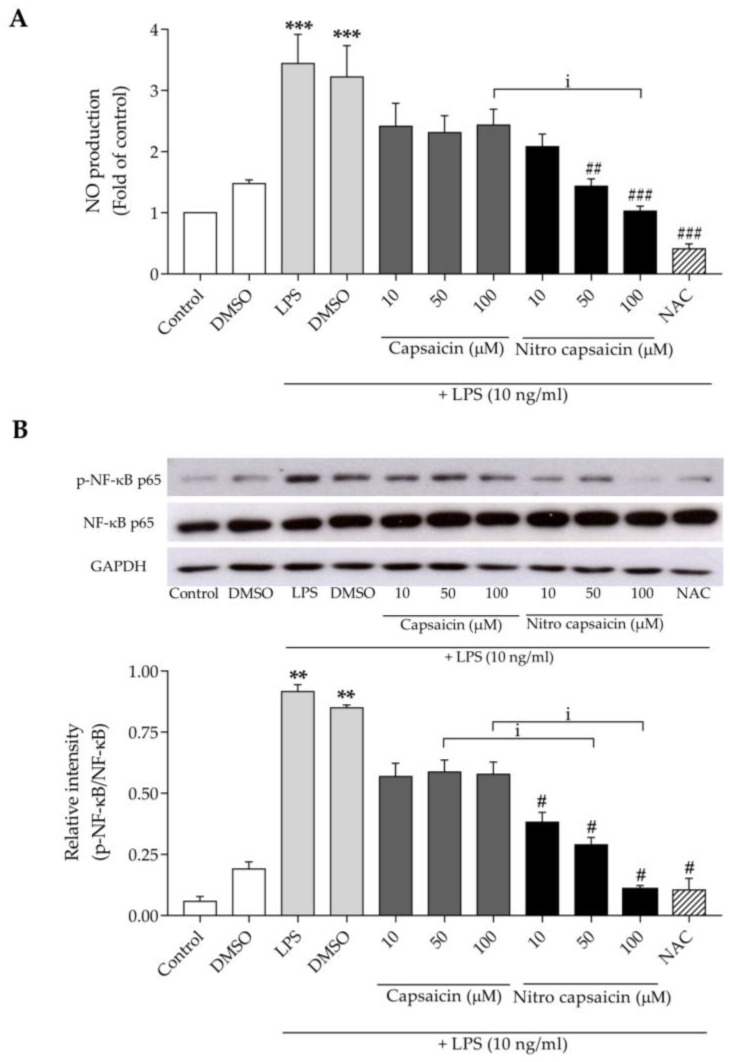
Effect of nitro capsaicin against LPS-induced NO production in SIMA9 cells. (**A**) Inhibition of LPS-induced NO production by nitro capsaicin; (**B**) Western blot analysis of p-NF-κB p-65 Ser536 and NF-κB p-65 expression. NAC (10 mM) was used as the positive control. ** p < 0.01 and *** p < 0.001 versus control group; *^#^ p <* 0.05, *^##^ p <* 0.01, and *^###^ p <* 0.001 versus LPS-treated group; and ^i^ *p* < 0.05 versus capsaicin-treated group.

Elevated nitric oxide (NO) has been reported to be a common pathological etiology of several neurodegenerative diseases [31,32]. Previous studies have reported that chronic microglial activation promotes a greater production of NO, which can damage neurons [33,34]. To determine the anti-inflammatory effects of nitro capsaicin, we first investigated its effects on NO production using the Griess assay. As shown in Figure 2A, treatment with 10 ng/mL of LPS for 24 h significantly increased NO levels to 3.44 ± 0.47-fold of the control group. Conversely, pretreatment with nitro capsaicin for 1 h strongly diminished the LPS-induced increase of NO levels in a concentration-dependent manner. The original capsaicin compound showed the trend of decreasing NO production, but the decreased NO levels were not significantly different compared to LPS treatment alone. These results suggested that nitro capsaicin at 100 µM counteracted LPS-induced NO production more potently than capsaicin did. As expected, N-Acetylcysteine (NAC) suppressed NO production [35,36]. Moreover, treatment with nitro capsaicin for 1 h significantly decreased the protein expression levels of inducible nitric oxide synthase (iNOS), which is a key enzyme for the synthesis of NO [37], compared to LPS treatment alone for 24 h (Supplementary Figure S1).

NF-κB is a key transcription factor of several genes involved in inflammatory responses during neurodegeneration, including the production of NO, cytokines, and chemokines [38]. We asked if nitro capsaicin could inhibit the NF-κB activation by measuring the phosphorylation of NF-κB p65 Ser536 and NF-κB p65 using western blot analysis. As shown in Figure 2B, after treatment with LPS at 10 ng/mL for 30 min, the phosphorylation of NF-κB p65 Ser536 was significantly increased relative to the control. However, pretreatment with nitro capsaicin for 1 h significantly decreased the LPS-induced phosphorylation of NF-κB p65 in a concentration-dependent manner. Noted, this effect was more robust than those of capsaicin pretreatment at 50 and 100 µM. These findings suggested that nitro capsaicin might mitigate microglial activation induced by LPS via suppressing NO production and that it is involved with NF-κB signaling.

### 3.3. Nitro Capsaicin Suppressed TNF-α, IL-1β, and IL-6 Production in SIMA9 Cells

**Figure 3:**
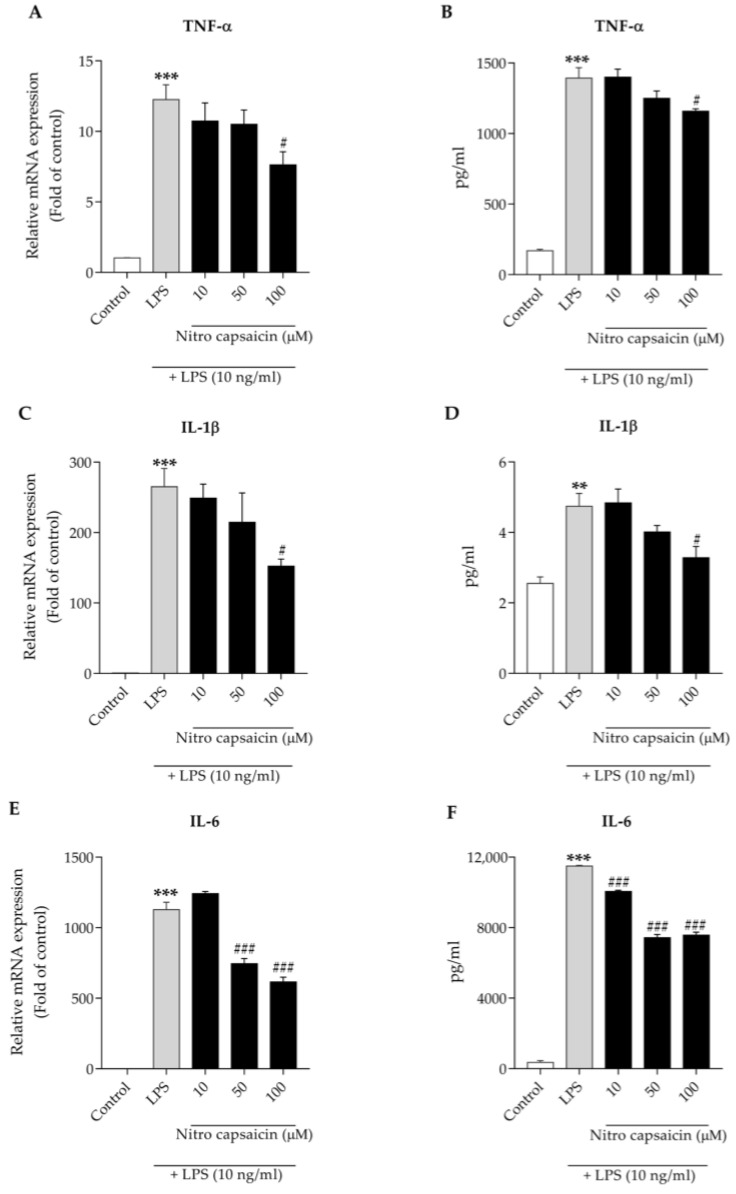
Effects of nitro capsaicin against LPS-induced proinflammatory cytokine production in SIMA9 cells. Nitro capsaicin decreased mRNA expression of (**A**) TNF-α, (**C**) IL-1β, and (**E**) IL-6. Nitro capsaicin reduced release of the cytokines (**B**) TNF-α, (**D**) IL-1β, and (**F**) IL-6. The control was DMSO treatment. ** p < 0.01 and *** p < 0.001 versus control group; *^#^ p <* 0.05 and *^###^ p <* 0.001 versus LPS-treated group.

Proinflammatory cytokines released from chronically activated pathological microglia are neurotoxic. Previous studies have reported that the upregulation of proinflammatory cytokines, especially TNF-α, IL-1β, and IL-6, can directly drive cytotoxicity in several cell types in the brain [10,11,12]. Here, we further determined the anti-inflammatory properties of nitro capsaicin by evaluating its regulatory effects on the expression of several proinflammatory factors using RT-PCR. We found that treatment with 10 ng/mL of LPS for 6 h caused an increase in the gene expression of proinflammatory cytokines, such as TNF-α, IL-1β, and IL-6. The incubation of nitro capsaicin significantly blunted mRNA expression of these cytokines at varying degrees (Figure 3A,C,E). Next, we measured the secretion of these cytokines using Milliplex ELISA. Consistent with transcripts, pretreatment with nitro capsaicin for 1 h inhibited TNF-α, IL-1β, and IL-6 released in microglial cells stimulated with LPS for 24 h (Figure 3B,D,F). These results indicated that nitro capsaicin exerts its anti-inflammatory activities by suppressing microglial activation, thereby reducing the production of proinflammatory cytokines.

### 3.4. Nitro Capsaicin Enhanced IL-4 and IL-10 Production in SIMA9 Cells

**Figure 4:**
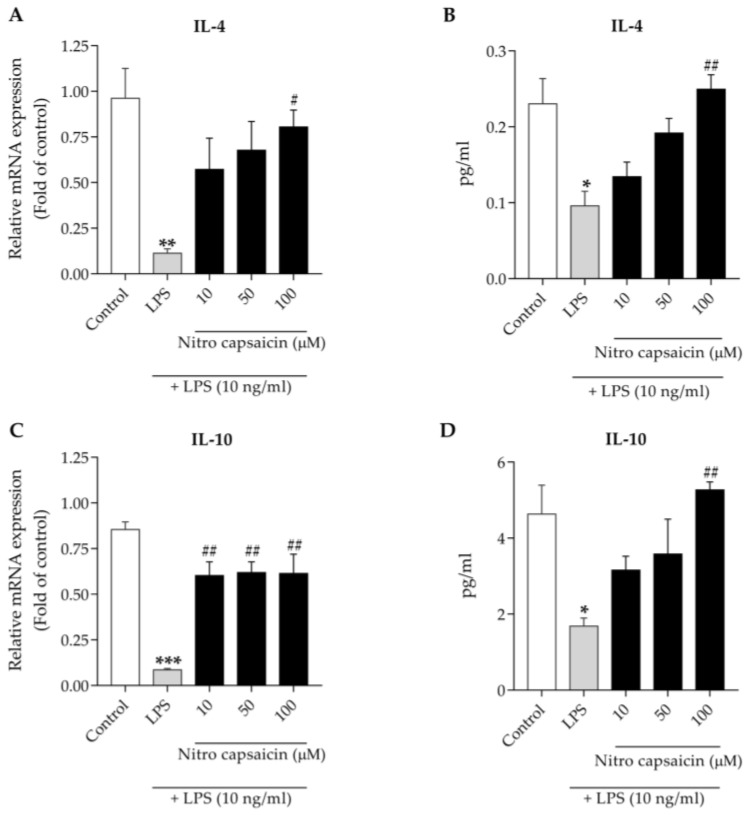
Effects of nitro capsaicin against LPS-suppressed anti-inflammatory cytokine production in SIMA9 cells. Nitro capsaicin increased mRNA expression of (**A**) IL-4 and (**C**) IL-10. Nitro capsaicin enhanced release of the cytokines (**B**) IL-4 and (**D**) IL-10. The control was DMSO treatment. * p < 0.05, ** p < 0.01, and *** p < 0.001 versus control group; *^#^ p <* 0.05 and *^##^ p <* 0.01 versus LPS-treated group.

Anti-inflammatory cytokines play a critical role in neutralizing pathologic neuroinflammation. Several studies reported that reductions in IL-4 and IL-10 production markedly worsened cognitive impairment in AD patients and in the hippocampus of the Aβ_1–42_-injected rat model [39,40]. A previous study demonstrated that the suppression of microglial activation by several agents, such as Betulinic acid, Pioglitazone, Deferoxamine, etc., alleviates neuroinflammation by enhancing the production of anti-inflammatory factors [41]. To investigate the effects of nitro capsaicin exposure on the production of anti-inflammatory cytokines, we first evaluated the mRNA expression levels of IL-4 and IL-10. We found that treatment with LPS at 10 ng/mL for 6 h markedly reduced mRNA expression of IL-4 and IL-10. However, the expression of these cytokines significantly increased when microglia were pretreated with nitro capsaicin for 1 h relative to LPS treatment alone (Figure 4A,C). Furthermore, LPS stimulation for 24 h decreased the expression of IL-4 and IL-10 at the protein level, but pretreatment with nitro capsaicin for 1 h upregulated both cytokines in a concentration-dependent manner (Figure 4B,D). Taken together, these results suggested that nitro capsaicin mediates anti-inflammatory effects on LPS-induced microglial activation via the induction of anti-inflammatory cytokines.

### 3.5. Nitro Capsaicin Exerted Anti-Inflammatory Effects and Suppressed NF-κB Activation in hCMEC/D3 Cells

**Figure 5:**
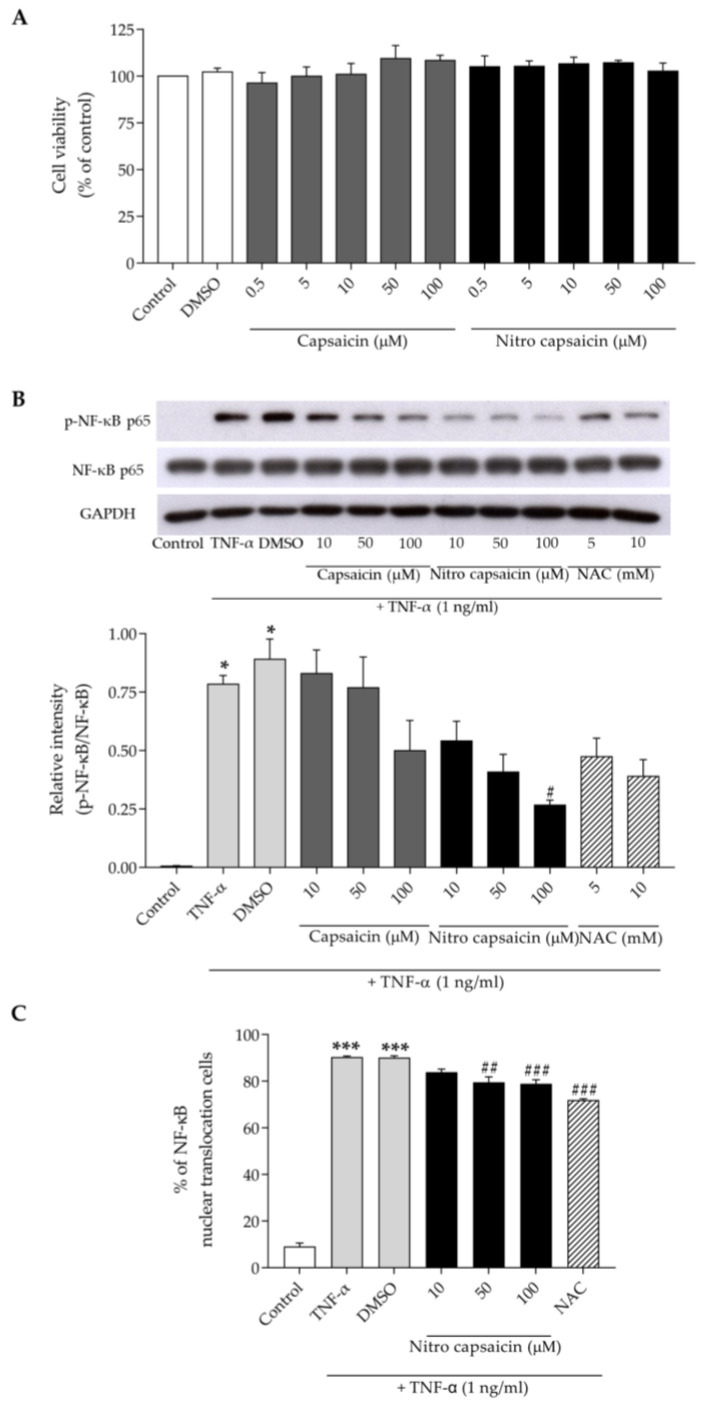
Effect of nitro capsaicin on NF-κB activation in hCMEC/D3 cells. (**A**) Cell viability following treatment with different concentrations of nitro capsaicin and capsaicin in hCMEC/D3 cells. (**B**) Representative western blot and quantification graph showing p-NF-κB p-65 Ser536 and NF-κB p-65 expression levels. (**C**) Graph represents percentage of NF-κB nuclear translocation induced by TNF-α in the absence or presence of nitro capsaicin. NAC (10 mM) was used as the positive control. * p < 0.05 and *** p < 0.001 versus control group; *^#^ p <* 0.05, *^##^ p <* 0.01, and *^###^ p <* 0.001 versus TNF-α-treated group.

To estimate the anti-inflammatory effects of nitro capsaicin on human brain endothelial cells, we initially examined nitro-capsaicin-induced cytotoxicity of hCMEC/D3 cells using an MTT assay. The cells were treated with nitro capsaicin or capsaicin at the indicated concentrations (0.5, 5, 10, 50, and 100 µM) for 24 h in serum-free conditions. We found that treatment with nitro capsaicin or capsaicin at all concentrations did not cause cytotoxicity (Figure 5A). Based on this result, a range of concentrations of both compounds (10–100 µM) was selected to evaluate possible anti-inflammatory effects against TNF-α-induced brain capillary endothelial damage. Previous studies reported that TNF-α can promote brain microvascular endothelial inflammation by activating several inflammatory pathways, including NF-κB [42,43]. Therefore, we sought to determine how nitro capsaicin affects brain endothelial inflammation that has been induced by TNF-α and suppressed NF-κB activation. To test this, we utilized western blot analysis to detect p-NF-κB p65 Ser536 and NF-κB p65 expression. As shown in Figure 5B, treatment with TNF-α at 1 ng/mL for 30 min significantly increased the p-NF-κB p65/NF-κB p65 ratio compared to the control group. However, the phosphorylation of NF-κB p65 was concentration-dependently diminished when cells were pretreated with nitro capsaicin (100 µM) for 1 h. This effect was not significantly different when compared between nitro capsaicin and capsaicin treatment. Moreover, we determined whether nitro capsaicin can suppress NF-ĸB translocation into the nucleus after 1 ng/mL TNF-α stimulation using the immunofluorescence staining assay. As shown in Figure 5C and Supplementary Figure S2, we found that pretreatment with nitro capsaicin for 1 h significantly reduced NF-ĸB nuclear translocation. These results illustrated that the anti-inflammatory effects of nitro capsaicin against TNF-α-induced brain capillary endothelial inflammation may be mediated via the regulation of NF-κB activation.

### 3.6. Nitro Capsaicin Inhibited Proinflammatory Cytokine Production in hCMEC/D3 Cells

**Figure 6:**
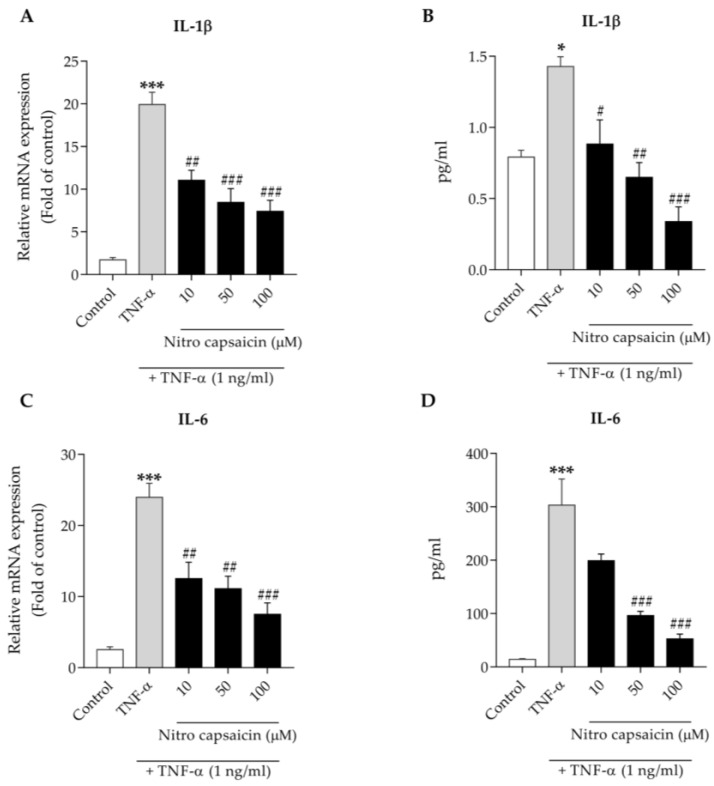
Effects of nitro capsaicin against TNF-α-induced proinflammatory cytokine production in hCMEC/D3 cells. Nitro capsaicin decreased mRNA expression of (**A**) IL-1β and (**C**) IL-6. Nitro capsaicin reduced release of the cytokines (**B**) IL-1β and (**D**) IL-6. The control was DMSO treatment. * p < 0.05 and *** p < 0.001 versus control group; *^#^ p <* 0.05, *^##^ p <* 0.01, and *^###^ p <* 0.001 versus TNF-α-treated group.

Several proinflammatory factors, such as cytokines and chemokines, released from damaged brain capillary endothelial cells, can drive neuroinflammation [13,14,15,16,17]. Thus, we determined the effects of nitro capsaicin on the production of TNF-α-stimulated proinflammatory cytokines IL-1β and IL-6. IL-1β and IL-6 mRNA expression were first evaluated using the RT-PCR assay. TNF-α treatment at 1 ng/mL for 6 h increased the mRNA levels of IL-1β and IL-6. However, cells pretreated with nitro capsaicin for 1 h had a significantly reduced expression of IL-1β and IL-6 mRNA in a concentration-dependent manner (Figure 6A,C). In addition, we investigated the nitro capsaicin pretreatment-mediated effects on cytokine release using the Milliplex ELISA assay. We found that the secretion of IL-1β and IL-6 was increased in hCMEC/D3 cell cultures treated with 1 ng/mL of TNF-α for 24 h. However, pretreatment with nitro capsaicin for 1 h significantly inhibited the release of IL-1β and IL-6 relative to TNF-α treatment alone (Figure 6B,D). Given these findings, we concluded that nitro capsaicin may prevent brain capillary endothelial inflammation from TNF-α-induced cell toxicity.

## 4. Discussion

In the present study, we demonstrated that nitro capsaicin significantly inhibited microglial activation by alleviating the production of proinflammatory factors and enhancing anti-inflammatory mediators in LPS-induced microglial activation in SIMA9 cells. Similarly, nitro capsaicin suppressed proinflammatory cytokines in hCMEC/D3 cells, thereby preventing brain capillary endothelial inflammation induced by TNF-α.

Our study demonstrated that nitro capsaicin more effectively induced anti-inflammatory properties than the original compound (capsaicin). With the same concentrations tested, nitro capsaicin significantly decreased NO production through the inhibition of iNOS expression and suppressed activation of NF-κB in microglia induced by LPS. Previous studies have reported that capsaicin exerts anti-inflammatory properties through TRPV1-dependent activation [44,45]. Thus, the action of modified capsaicin (nitro capsaicin) may be elicited through its binding with TRPV1. This hypothesis is supported by a recent study showing that nitro capsaicin, modified by the replacement of NO_2_ at OCH_3_ on the aromatic region, increased the negative charge and the dipole moment, which contributed to enhanced π-stacking interactions. The enhanced π-stacking interactions are indicative of the binding affinity value of nitro capsaicin being higher than that of capsaicin at the binding pocket of the TRPV1 channel [25]. Therefore, the improved anti-inflammatory properties of nitro capsaicin compared to its parent compound might be due to its higher affinity for the TRPV1 channel. However, further study is required to prove this hypothesis.

Moreover, nitro capsaicin inhibited the LPS-mediated production of proinflammatory cytokines, such as TNF-α, IL-1β, and IL-6. It also increased the mRNA expression and release of the anti-inflammatory cytokines IL-4 and IL-10 in LPS-stimulated SIMA9 cells. Previous studies have reported that during neuroinflammation, microglial cells switch from the M2 anti-inflammatory stage to the M1 proinflammatory stage, ultimately driving disease progression [46,47]. Capsaicin has been shown to shift M1 microglia to M2 microglia, as demonstrated by the decreasing expression of M1 proinflammatory markers and the increasing M2 anti-inflammatory markers against LPS-damaged substantia nigra [20]. Here, we showed that nitro capsaicin can decrease the production of proinflammatory cytokines and enhance anti-inflammatory cytokines. Therefore, nitro capsaicin may be able to regulate the microglia polarization shift from M1 to M2 stages in activated microglia. However, future studies determining the microglial phenotype are required to prove this hypothesis.

Here, we showed that nitro capsaicin likely elicits its anti-inflammatory effects through the NF-κB signaling pathway. However, other TRPV1-related anti-inflammatory pathways have been shown to be modulated by capsaicin. For example, capsaicin exhibits strong antioxidant effects through the upregulation of the protein levels for antioxidant enzymes, such as catalase and SOD2 [48]. Additionally, capsaicin was shown to enhance catalase enzyme levels via the upregulation of Nrf2 [49]. Furthermore, capsaicin has been shown to stimulate PPARγ, which causes the suppression of inflammation during liver fibrosis [49,50]. The greater effects of nitro capsaicin against microglial activation may be regulated by any of these TRPV1-related anti-inflammatory pathways, which require further investigation.

Taken together, we showed that nitro capsaicin can exert potent anti-inflammatory properties on microglial activation and brain microvascular endothelial inflammation. A previous study showed that capsaicin can pass the blood-brain barrier very easily. Specifically, the authors observed measurable capsaicin levels in rat brain sections after its intravenous and subcutaneous administration [51]. Whether nitro capsaicin can enter the brain remains to be tested. The structure of nitro capsaicin is composed of the hydrocarbon chain in their tail section, which enhances its hydrophobic property. This likely facilitates the entrance of the compound into the cells [52]. Therefore, it is possible that nitro capsaicin might be permeable to brain endothelial cells. Given the data presented here, we proposed that nitro capsaicin could be beneficial in mitigating the inflammatory response in the brain. Further studies are essential for determining its effect on the brain in vivo.

## 5. Conclusions

Capsaicin has been shown to exert powerful neuroprotective effects and to improve behavioral impairment in several neurodegenerative animal models. However, capsaicin’s irritant properties limit its clinical application. Here, we demonstrated that nitro capsaicin, a newly modified capsaicin with less pungent characteristics, acts as a more potent anti-inflammatory than capsaicin in both microglial and brain endothelial cell models. This effect is potentially due to the suppression of NF-κB activation. This evidence demonstrates the potential for the development of nitro capsaicin as an anti-neuroinflammatory agent. Further in vivo animal studies are crucial for investigating its effect on the brain.

## Data Availability

Not applicable.

## Supplementary Materials

The following supporting information can be downloaded at https://www.mdpi.com/article/10.3390/biomedicines10112680/s1: Figure S1: Effect of nitro capsaicin against LPS-stimulated iNOS expression; Figure S2: Effect of nitro capsaicin against TNF-α-induced NF-ĸB nuclear translocation; Figure S3: Original images for blots (Figure 2B); Figure S4: Original images for blots (Figure 5B); and Figure S5: Original images for blots (Supplementary Figure S1).
