# Supplementary material for: Nitro Capsaicin Suppressed Microglial Activation and TNF-α-Induced Brain Microvascular Endothelial Cell Damage
Source: Biomedicines. 2022 Oct 23;10(11):2680. doi: 10.3390/biomedicines10112680 (PMC9687943; doi:10.3390/biomedicines10112680)
Supplement: Supplementary file 1 [file biomedicines-10-02680-s001.zip › biomedicines-1937834-supplementary.pdf]

biomedicines

MDPI

Supplementary Materials: Nitro Capsaicin Suppressed Microglial Activation and TNF-α-Induced Brain Microvascular Endothelial Cell Damage

Sopana Jamornwan, Tanida Chokpanuwat, Kwanchanok Uppakara, Thanet Laorob, Uthai Wichai, Pimonrat Ketsawatsomkron and Witchuda Saengsawang

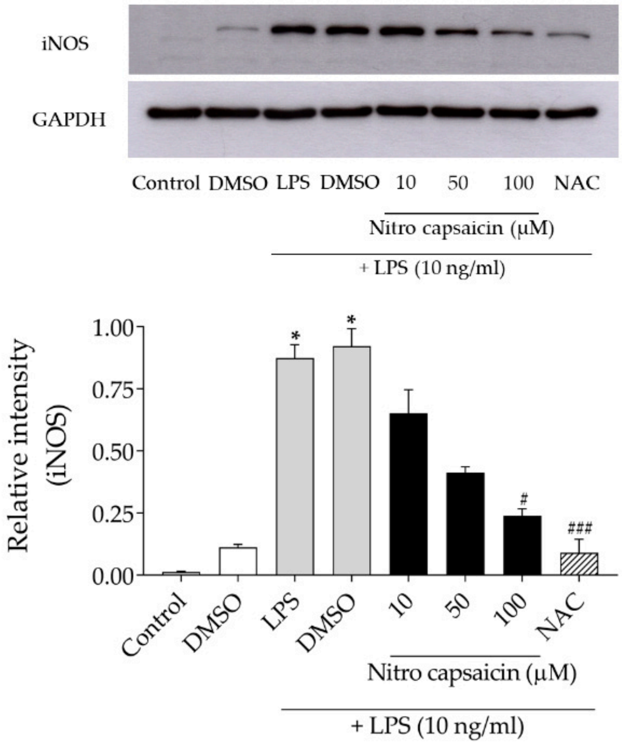

Figure S1. Effect of nitro capsaicin against LPS-stimulated iNOS expression. SIMA9 cells were pretreated for 1 h with nitro capsaicin before being exposed to 10 ng/ml LPS for 24 h followed by measurement iNOS protein expression levels using western blot assay. NAC (10 mM) was used as the positive control. $^{*}p < 0.05$ compared to control (untreated cells); $^{\#}p < 0.05$ and $^{\#\#\#}p < 0.001$ compared to LPS-treated cells (One-way ANOVA followed by Tukey's test).

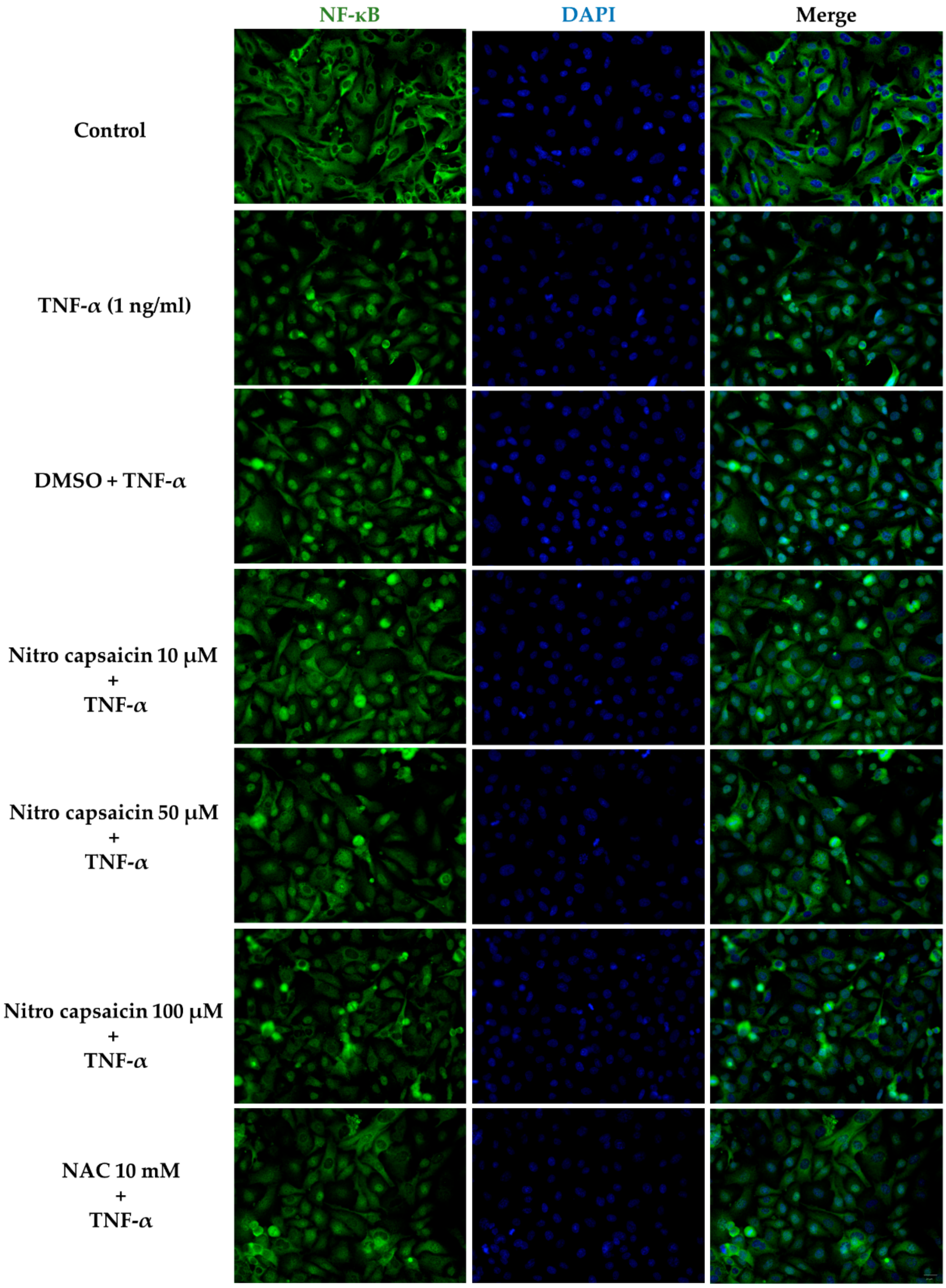

Figure S2. Effect of nitro capsaicin against TNF-α-induced NF-κB nuclear translocation. hCMEC/D3 cells were treated with nitro capsaicin for 1 h in the presence or absence of TNF-α at 1 ng/ml for 30 min. The activation of NF-κB nuclear translocation was evaluated by staining for NF-κB (green) and nucleus (blue), and imaged under fluorescence microscope (Scale bar = 20 μm).

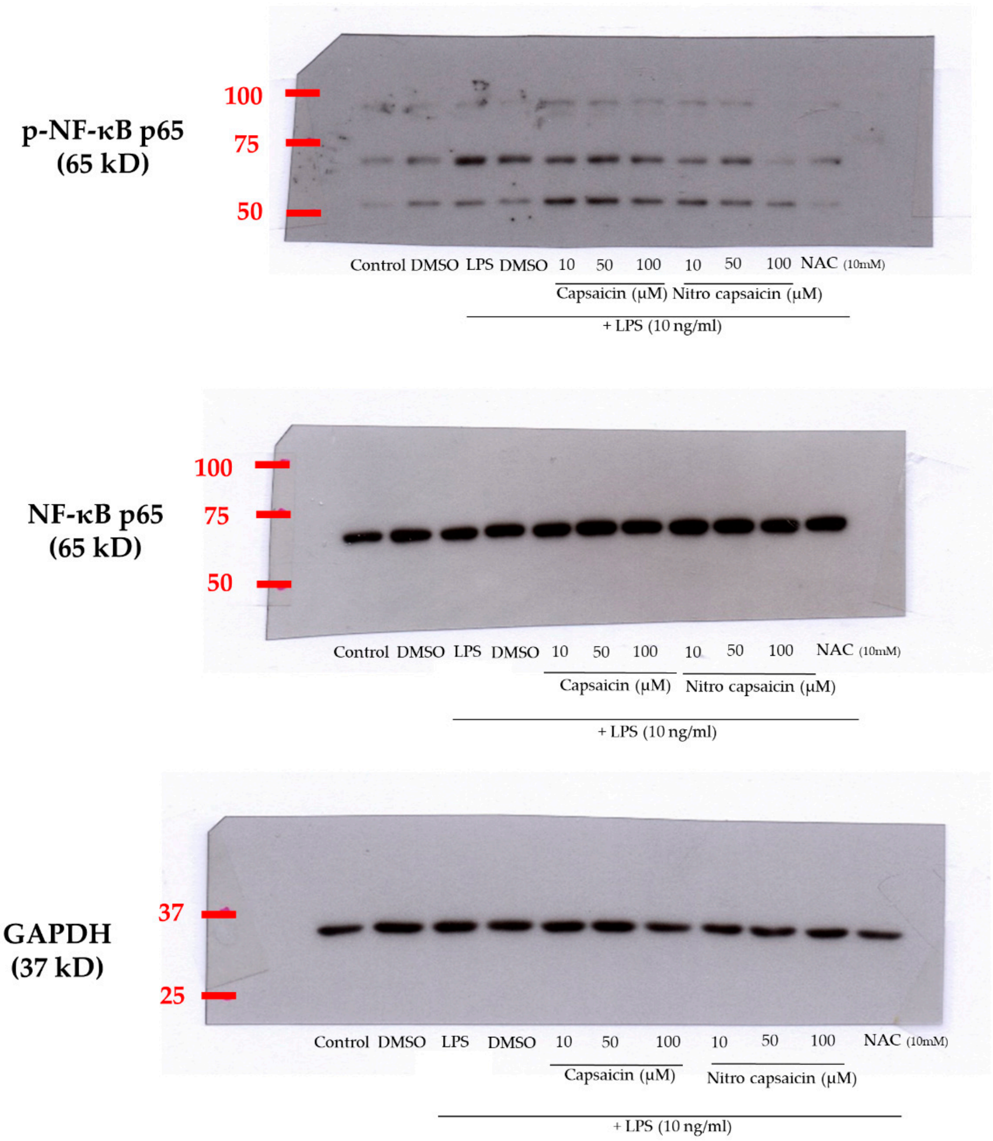

Figure S3. Original images for blots (Figure 2B).

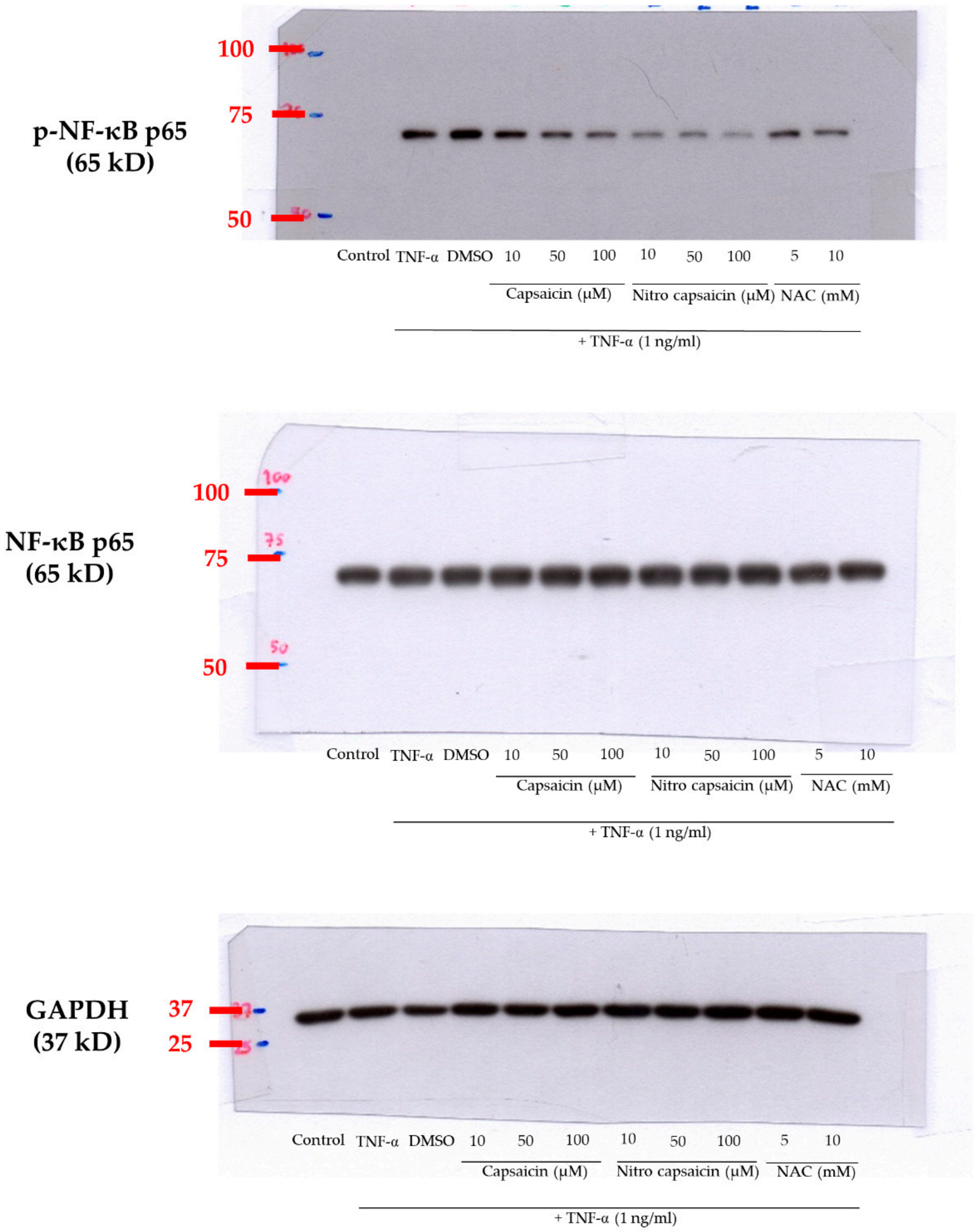

Figure S4. Original images for blots (Figure 5B).

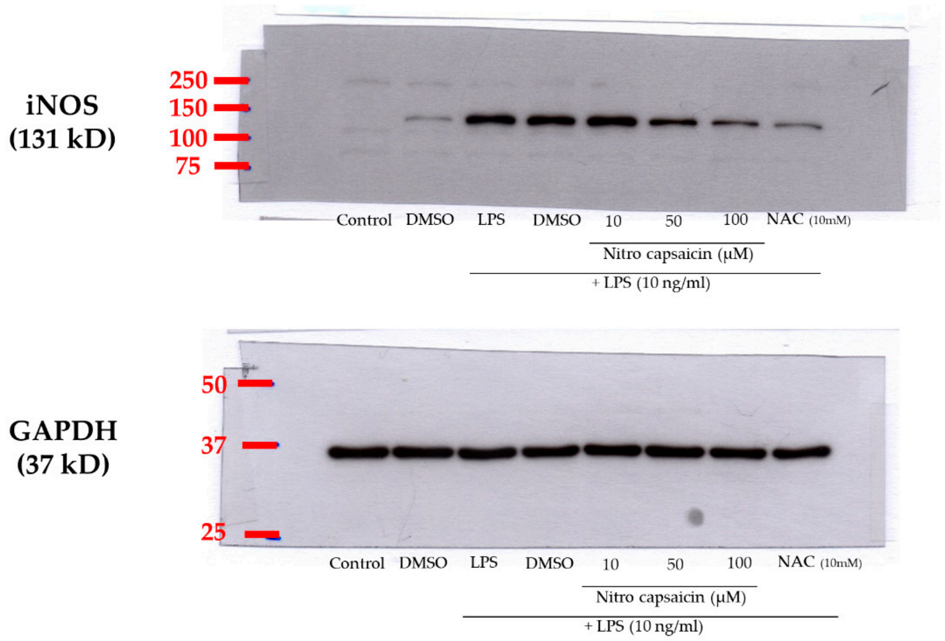

Figure S5. Original images for blots (Supplementary Figure S1).
